# Diffusion measurements of hydrocarbons in H-MCM-41 extrudates with pulsed-field gradient nuclear magnetic resonance spectroscopy†

**DOI:** 10.1039/d2cp00138a

**Published:** 2022-03-16

**Authors:** Vladimir V. Zhivonitko, Zuzana Vajglová, Päivi Mäki-Arvela, Narendra Kumar, Markus Peurla, Ville-Veikko Telkki, Dmitry Yu. Murzin

**Affiliations:** NMR Research Unit, University of Oulu Oulu Finland; Åbo Akademi University, Johan Gadolin Process Chemistry Centre Henriksgatan 2, Turku/Åbo 20500 Finland dmurzin@abo.fi; Institute of Biomedicine, University of Turku Kiinamyllynkatu 10 Turku 20520 Finland

## Abstract

Mesoporous materials are promising catalysts for production of biofuels. Herein, H-MCM-41 catalysts with different concentrations of the silica Bindzil binder (10–50 wt%) were prepared and characterized using pulsed-field gradient (PFG) NMR in the powder form and as extrudates. Effective diffusion coefficients (*D*_e_) are measured in all cases. Diffusivities of *n*-hexadecane were found smaller for extrudates as compared to the powder catalysts. The estimates of diffusive tortuosity were also determined. PFG NMR data showed one major component that reveals diffusion in interconnected meso- and micropores and one other minor component (1–2%) that may correspond to more isolated pores or may represent complex effects of restricted diffusion. Therefore, several approaches including initial slope analysis of spin-echo attenuation curves, two-component fitting and Laplace inversion were used to discuss different aspects of diffusional transport in the studied H-MCM-41 materials. Correlations between *D*_e_ and the amount of Bindzil, the specific surface area, the micropore volume, the particle size, the total acid sites and the Lewis acid sites are discussed.

## Abstract 2

Diffusivities of *n*-hexadecane were measured using pulsed-field gradient (PFG) NMR for extrudates and powder catalysts comprising H-MCM-41′ and silica Bindzil binder.

## Graphical abstract

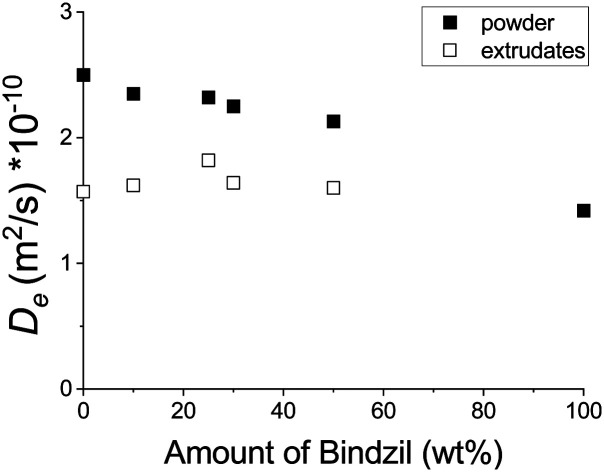

## 1. Introduction

Mesoporous catalysts, such as metal modified H-MCM-41 are promising for production of biofuels and have been used in hexadecane hydrocracking.^1^ The latter reaction is industrially performed in a continuous reactor, for which catalyst extrudates are used. A need to employ mm sized shaped catalyst bodies in industrial reactors implies that in addition to intrinsic kinetics, transport phenomena and reactor engineering constitute an essential part of catalytic engineering. Especially relevant is the mass transfer inside the porous catalyst particles/layers, as well external diffusion in the laminar film surrounding the catalyst particles. Elucidation of the role of internal diffusion requires understanding how mass transfer occurs in shaped catalyst bodies, made typically by extrusion, exhibiting a distribution of pores of different types. To understand the role of mass transfer limitations in porous catalyst particles, knowledge of diffusivities of different feedstock in porous media is thus essential. Assessment of the impact of mass transfer in shaped catalyst bodies was done for example by studying the gravimetric uptake of 2,2-dimethylbutane in extrudates formed from clay binders and hierarchical zeolites of MFI type.^2,3^ The latter are prepared through introduction of mesoporosity to microporous materials by, *e.g.*, desilication.

Pulsed-field gradient (PFG) NMR spectroscopy is in this context a valuable tool to determine apparent diffusivities of a fluid in a constrained catalyst matrix. It has been recently applied to investigate diffusivities of C4-C8 hydrocarbons in large NaX zeolites,^3,4^ hexane in pseudomorphic MCM-41,^5^ heptane in mesoporous ZSM-5,^6^ heptane and pentadecane in H-Beta zeolite Bindzil extrudates,^7^ octane in micro- and mesoporous USY,^8^ cyclohexane in SBA-15 sulfonic acids,^9^ and also hexadecane in mesoporous MCM-41.^10^ Structural properties of a porous material can be determined by measuring the relationship between diffusion in a confined environment and in a bulk, *D*/*D*_0_.^11^ PFG NMR is also a superior method for analysing pore connectivity in complex porous solids^9^ and, *e.g.*, limited diffusion of large molecules in dealuminated, mesoporous zeolite was observed when non-ordered mesopores were present.^8^

Some recent examples of PFG NMR utilization in catalysis include investigation of grafting zirconia on alumina as a support for silicotungstic acid revealing that grafting leads to a more tortuous structure.^12^ Another example is related to bimetallic AuPt/C catalysts reporting presence of two distinct diffusion regimes with the values of self-diffusivities differing by more than an order of magnitude.^13^ Apparently self-diffusivity in the slow diffusion regime was assigned to diffusion within micropores being most affected by deactivation. Pore diffusion behaviour was also explored with an aid of PFG NMR for hierarchical alumina, for which macropores were introduced to the mesoporous structures.^14^

In this work, PFG NMR was used as a tool to determine diffusivity of *n*-hexadecane in H-MCM-41 powder and extrudate catalysts containing different amounts of Bindzil as a binder. PFG NMR experiments were performed using a stimulated echo pulse sequence with bipolar gradients.^15^ Three different methods were used to analyse the acquired data, including extracting averaged apparent diffusion coefficients from the initial slopes of spin-echo attenuation curves, two-component fitting analysis, and applying the Laplace inversion procedure. The results provide versatile information about apparent diffusion coefficients. Altogether, this gives information about the diffusion mechanism, pore blockage and interconnectivities. In addition, the correlation between catalyst properties and diffusivities of hexadecane are discussed together with effective diffusion coefficient1
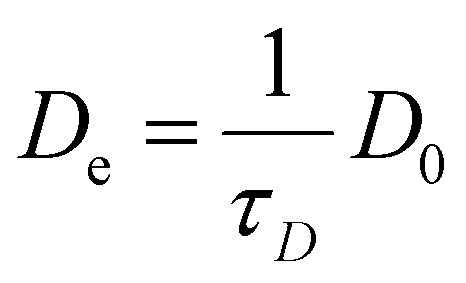
which is related to catalytic properties. In eqn (1)*D*_0_ is the bulk self-diffusivity and *τ*_*D*_ is diffusive tortuosity.^7,16,17^ Note that the tortuosity as defined in eqn (1) can also depend on the chemical nature of the diffusing molecules, if the latter have functional groups interacting with the solid phase.^18^

As H-MCM-41 porous materials have complex structure consisting of multitude of pore structure levels (extrudate → grain → particle → mesopores → micropores), the dependence of *τ*_*D*_ on the properties of materials is complex.^19^ Apart from few attempts to quantify the parameter *τ*_*D*_ (or its inverse *ξ* = *τ*^−1^_D_) for extruded catalysts,^7^ quantification of *τ*_*D*_ for shaped catalysts, such as for extrudates or pellets, is lacking. Such materials contain not only micro- and mesopores, but also macropores by virtue of their preparation, enhancing thereby the transport properties in the longitudinal direction. In the current work the extrudates were prepared with different concentrations of the silica binder to explore what would be the influence of the binder on the effective diffusion coefficients (*D*_e_) providing thereby information not currently available. Moreover, *n*-hexadecane, a typical probe molecule emulating diesel was used in PFG NMR diffusion measurements.

## 2. Experimental

### 2.1 Preparation of the catalysts

Shaping of catalysts by extrusion typically cannot be done without binders, and other additives ensuring proper rheological properties, preventing agglomeration, creating transport pores and improving mechanical stability. In the current work, extrusion was done for the mesoporous material H-MCM-41 *per se* as well as for more realistic from the industrial viewpoint cases when application of a binder is required. Extrusion in practice is done from suspensions containing various additives (binders, porogens, rheology improvers, peptizers, etc). The strategy for catalyst preparation by extrusion, adopted by the authors previously,^20^ included along other recipes, preparation of a mechanical mixture of the binder and the catalytic phase by mechanical mixing, drying, calcination and grinding. This final mixture in a powder form, which already experienced mechanical impact leading to alteration of physico-chemical properties, underwent extrusion.

In the current work, eleven samples were thus prepared, namely: pristine H-MCM-41 mesoporous catalytic material in the powder form and as extrudates; the pristine powder sample of the Bindzil binder (Bindzil-50/80, 50% colloidal SiO_2_ in H_2_O from Akzo Nobel) prepared by evaporation of an aqueous suspension at 40 °C under vacuum and drying at 100 °C for 7 h; and four H-MCM-41 catalysts with a different content of the Bindzil binder (10–50 wt%) prepared both in the powder form and as extrudates.

It should be also considered that binders, having their major role in achieving the desired mechanical stability, in a general case might adversely influence the catalytic behavior. Therefore, when there is no need to dilute the overreacting active phase, the amount of the binder should not be too large. Subsequently in the current work, different amounts of binders were studied to explore the influence of binders on diffusional properties.

MCM-41 catalyst was synthesized from a gel solution at 100 °C for 72 h. The gel solution itself was prepared from fumed silica (16.6 g, BDH Laboratory), sodium silicate solution (22.8 g, Merck), cetyltrimethylammonium bromide (51.8 g, Sigma-Aldrich), aluminium isopropoxide (4 g, Sigma-Aldrich) and tetramethylammonium silicate (46.8 g, Sigma-Aldrich), treated with ultrasound at 80 Hz and 100 W for 8 h. The H-MCM-41 proton form was transformed from Na-MCM-41 by ion exchange with 0.5 M ammonium chloride solution. Subsequently, H-MCM-41 was dried and calcined in a step calcination procedure: 25 °C – 3 °C min^−1^ – 250 °C (held for 1 h) and 250 °C – 6.6 °C min^−1^ – 550 °C (held for 6 h). Details are provided in ref. 20, 21.

In the case of catalysts containing both materials, a colloidal silica Bindzil binder (10–50 wt%) in the suspension form was added directly into the suspension of grinded H-MCM-41. The suspension was stirred at 50 rpm and ambient temperature for 24 h. In the subsequent steps, water was evaporated at 40 °C under vacuum, followed by drying at 100 °C for 7 h and calcination at 500 °C for 4 h.^22^ All powder samples were grinded and sieved into a fraction below 63 μm.

Extrudates were prepared using the weight ratio of the catalyst/water/methylcellulose of 33/65/2 as a suspension for extrusion and catalyst shaping.^20,21^ The extrudates were shaped in the one-screw extrusion device (TBL-2, Tianjin Tianda Beiyang Chemical Co. Ltd., China) into the cylindrical shapes with a diameter of 1.5 mm. After drying (at 110 °C for 7 h) and calcination (at 500 °C for 4 h), the extrudates were cut to a length of *ca.* 10 mm.

The samples were named using capital letters referring to their components (B = Bindzil; M = H-MCM-41), numbers referring to the weight percentage of the component and a letter referring to the form of the sample (E = extrudate; P = powder). For example, B10M90-P refers to the sample including 90% of H-MCM-41 and 10% of Bindzil in powder form. The compositions of all eleven samples studied here are listed in Table 1.

**Table 1:** Catalyst characterization data

Catalyst	Sample	A	*V*_p_	*V*_μ_	*V*_m_	*d*_p_	*V*_m_/*V*_μ_	TAS	BAS	LAS	B/L	*d*_TEM_	*d*_SEM_
*—*	*—*	m^2^ g^−1^	cm^3^ g^−1^	cm^3^ g^−1^	cm^3^ g^−1^	nm	—	μmol g^−1^	μmol g^−1^	μmol g^−1^	*—*	nm	nm
Bindzil (P)	B100-P	157^a^	0.30	0.01	0.29	2.9	29	2	1	1	1.0	30	47
H-MCM-41 (P)	M100-P	797	0.86	0.28	0.58	1.0	2	140	84	56	1.5	37	85
H-MCM-41 (E)	M100-E	678	0.89	0.18	0.71	1.0	4	98	55	43	1.3	32	84
H-MCM-41 with 10% Bindzil (P)	B10M90-P	662	0.90	0.16	0.74	1.1	5	78	45	33	1.4	33	82
H-MCM-41 with 10% Bindzil (E)	B10M90-E	647	0.84	0.17	0.68	1.0	4	71	36	34	1.1	40	76
	B10M90-T	733	0.80	0.25	0.55	—	2	127	76	51	1.5	—	—
H-MCM-41 with 25% of Bindzil (P)	B25M75-P	430	0.52	0.11	0.41	1.0	4	75	41	34	1.2	34	82
H-MCM-41 with 25% of Bindzil (E)	B25M75-M	427	0.53	0.10	0.43	1.0	4	78	42	36	1.2	35	70
	B25M75-T	637	0.72	0.21	0.51	—	2	106	63	42	1.5	—	—
H-MCM-41 with 30% of Bindzil (P)	B30M70-P	556	0.72	0.20	0.52	1.0	3	57	28	30	0.9	44	111
H-MCM-41 with 30% of Bindzil (E)	B30M70-E	500	0.70	0.18	0.52	1.0	3	56	31	26	1.2	40	76
	B30M70-T	605	0.69	0.20	0.49	—	2	99	59	40	1.5	—	—
H-MCM-41 with 50% Bindzil (P)	B50M50-P	379	0.55	0.10	0.45	1.0	4	30	16	14	1.1	42	69
H-MCM-41 with 50% Bindzil (E)	B50M50-E	359	0.56	0.10	0.46	1.0	5	23	13	9	1.4	39	66
	B50M50-T	477	0.58	0.15	0.43	—	3	71	43	29	1.5	—	—

### 2.2 Characterization of the catalysts

All six powder catalysts and five extrudates were characterized in detail. The textural properties were analysed using nitrogen physisorption measurement on Micromeritics 3Flex-3500. The surface area, pore volume and pore size distribution were determined by the Dubinin-Radushkevich, and density functional theory (DFT) methods, respectively. For the mesoporous Bindzil binder, the Brunauer–Emmett–Teller (BET) method was used for calculations of the specific surface area. Morphological studies, the crystal particle sizes and their agglomerates were determined by scanning electron microscopy (SEM, Zeiss Leo Gemini 1530) and transmission electron microscopy (TEM, JEOL JEM-1400Plus). The amount of Brønsted and Lewis acid sites was quantified by Fourier transform infrared spectroscopy using pyridine as the probe molecule (ATI Mattson FTIR Infinity Series). The strength of the acid sites was classified based on the temperature at which pyridine desorbs from the catalyst, *i.e.*, desorption between 100–250 °C, 250–350 °C, and 350–450 °C was ascribed to weak, medium, and strong acid sites, respectively. The Brønsted acidity was quantified from the absorption band at 1550 cm^−1^ and the Lewis acidity from the adsorption band 1450 cm^−1^ using previously reported data of Emeis.^23^

### 2.3 PFG NMR experiments

PFG NMR experiments were performed at 25 °C on a Bruker 600 MHz spectrometer equipped with a 5 mm DiffBB BBO diffusion probe (maximum gradient *ca.* 17 T m^−1^). A stimulated echo pulse sequence with bipolar magnetic field gradient pulses was used for the diffusion measurements.^15,24^ The gradient pulses (400 μs) were followed by relatively long stabilization delays (1.4 ms) to avoid possible deleterious effects of the eddy currents due to gradient switching. Relatively long observation times Δ (20 and 240 ms) were used to assess the effective diffusion coefficients at the long diffusion time limit. Recycling delays and spin-echo times were optimized by estimating *T*_1_ and *T*_2_ relaxation times by inversion-recovery and CPMG measurements, respectively. The samples for the NMR analysis were prepared by immersing H-MCM-41 materials in pure *n*-hexadecane (>99%, Sigma-Aldrich) followed by evacuation of air bubbles from the materials and removing the excess solvent by rolling over a filter paper.

The experiments resulted in acquisition of a set of one-dimensional ^1^H NMR spectra of *n*-hexadecane measured with increasing gradient strengths (see, *e.g.*, Fig. S3a, ESI†). As expected, the NMR signals attenuated strongly due to diffusion as the gradient strength increased. The resulting signal amplitudes were converted into spin-echo attenuation curves (*e.g.*, Fig. 4b), *E*(*b*,Δ), by calculating *b* factors from the gradient amplitudes and time delays in the NMR experiments followed by normalizing the curve to unity at *b* → 0. The following equation was used to calculate the *b* factors in s m^−2^2
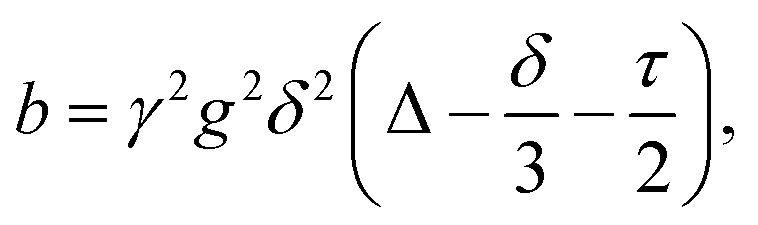
where *γ* = 4257.64 Hz G^−1^ is the ^1^H gyromagnetic ratio, *g* is the variable amplitude of applied gradient (G m^−1^), *δ* = 800 μs is twice the gradient pulse duration due to the use of the bipolar gradient, *τ* = 1.4407 ms is the time between gradient pulses in a bipolar gradient pulse pair and Δ is the variable diffusion (observation) time.^24^

The initial part of the echo attenuation curves was used to obtain apparent diffusion coefficients (*D*(Δ)), assuming that the *E*(*b*,Δ) is proportional to exp(–*D*(Δ)*b*) at the small *b* factor values which is equal to determining the second moment of the average diffusion propagator. *D*(Δ) coefficients were used in selected cases to estimate the surface-to-volume ratio (*S*/*V*) (see, *e.g.*, Fig. S3b, ESI†), following the approach developed by Mitra^25^ stating that at small Δ times the following holds3
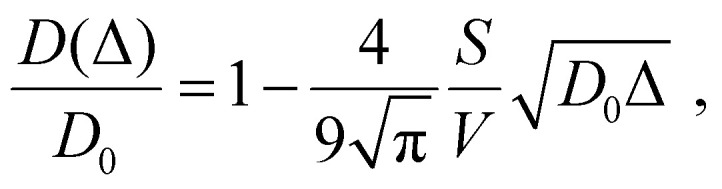
where *D*_0_ is the bulk diffusion coefficient. The *S*/*V* ratio was used to estimate the characteristic size of the restriction that could lead to the slight alteration of the apparent diffusion coefficients as shown in Fig. S3b (ESI†) assuming the spherical geometry as a first approximation, *i.e.*, *d*_gr_ = 6*V*/*S*.

In the further processing, the following equation was used to perform the two-component fitting analysis

4 *E*(*b*, *p*_1_, *p*_2_, *D*_1_, *D*_2_) = *p*_1_exp (−*D*_1_*b*) + *p*_2_exp (−*D*_2_*b*)

where *p*_1_ and *p*_2_ are populations of the fast (*D*_1_) and the slow (*D*_2_) diffusion components and *b* is gradient or diffusion weighting factors varying in the PFG NMR experiment.

In addition, the Laplace inversion procedure was performed using the approach developed by Teal and Eccles^26^ and applying exp(–*Db*) as a kernel.

## 3. Results and discussion

### 3.1 Catalyst characterization

**Fig. 1:**
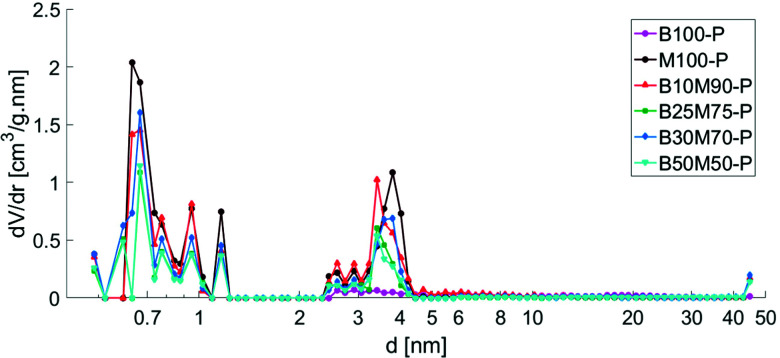
Pore size distribution of powder catalysts calculated by DFT method. Legend: Bindzil binder (B100-P),^7^ H-MCM-41 without binder (M100-P), H-MCM-41 with 10 wt% Bindzil (B10M90-P), H-MCM-41 with 25 wt% Bindzil (B25M75-P), H-MCM-41 with 30 wt% Bindzil (B30M70-P), H-MCM-41 with 50 wt% Bindzil (B50M50-P).

**Fig. 2:**
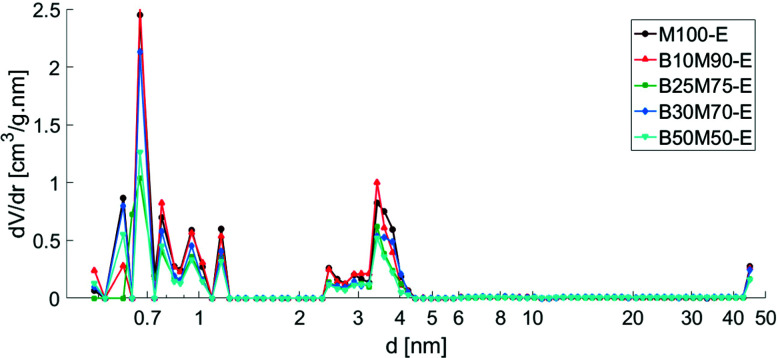
Pore size distribution of extrudates calculated by DFT method. Legend: H-MCM-41 without binder (M100-E), H-MCM-41 with 10 wt% Bindzil (B10M90-E), H-MCM-41 with 25 wt% Bindzil (B25M75-E), H-MCM-41 with 30 wt% Bindzil (B30M70-E), H-MCM-41 with 50 wt% Bindzil (B50M50-E).

**Fig. 3:**
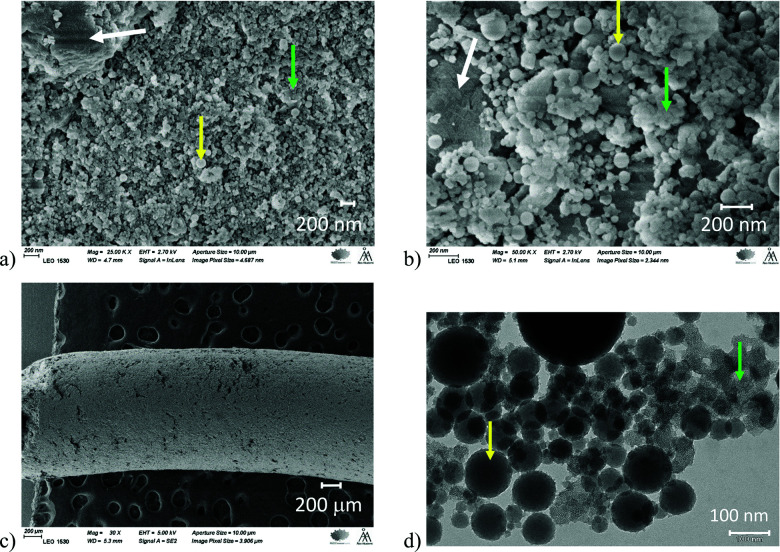
SEM images: (a) H-MCM-41 with 25 wt% Bindzil binder (B25M75-P, scale 200 nm); (b and c) H-MCM-41 with 50 wt% Bindzil binder (B50M50-E, scales 200 nm and 200 μm). TEM image: (d) H-MCM-41 with 50 wt% Bindzil binder (B50M50-E, scale 100 nm). H-MCM-41, Bindzil, and interfacial interaction of the phases are pointed green, yellow, and white arrows, respectively.

A purely mesoporous pore size distribution was observed for the Bindzil binder (Fig. 1 and ref. 7, 25) while H-MCM-41 catalyst and its agglomerates with a binder (Fig. 1 and 2) contained *ca.* 20–30 vol% of micropores from the total micro- and mesopore volume (Table 1). The specific surface area for the pristine powder H-MCM-41 catalyst was *ca.* five times higher than for the Bindzil binder. The measured specific surface areas of the H-MCM-41 catalysts with the Bindzil binder were *ca.* 10–30% lower than the theoretical values calculated from the contributions of non-agglomerated neat components in the powder form. As can be seen from Table 1 experimentally observed values are lower not only for the surface area, but also for the microporous volume, pointing out on blocking of the micropores. These deviations from the theoretical values are attributed to the catalyst synthesis procedure, in particular application of mechanical forces during preparation of the mechanical mixture of H-MCM-41 with the binder and subsequent shaping by extrusion. These operations resulted in a partial blockage of the micropore mouths, lower micropore volumes and grain boundary interactions between H-MCM-41 and the Bindzil binder. This is in line with the literature^7,20–22,27–33^ and SEM analysis (Fig. 3).

**Fig. 4:**
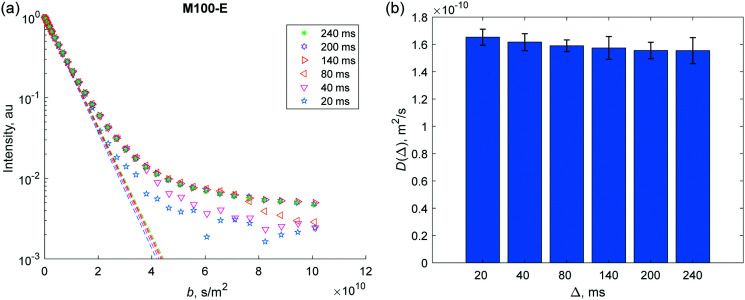
(a) Spin-echo signal attenuation curves at different diffusion times Δ (see legend) obtained for *n*-hexadecane in H-MCM-41 (M100-E) extrudate and representing the two-component like shape that was common for all H-MCM-41 samples. The straight lines show the initial slope of the curves. (b) Apparent diffusion coefficient values *D*(Δ) extracted from the initial slope data. Similar data for other samples are presented in the ESI.†

When comparing, for example, the mesoporous material MCM-41 in the form of extrudates with the powder from of the same material (Table 1) the changes imposed by the mechanical forces during extrusion *per se* are clearly visible. Note that the volume of micropores has decreased from 0.28 to 0.18 cm^3^ g^−1^, while the volume of mesopores has increased. Introduction of the binder to the mesoporous MCM-41 in low amounts (10%) diminished the surface area and increased the volume of mesopores already for the mechanical mixture prepared under the mechanical impact (stirring and grinding). Extrusion made thereafter changed only insignificantly the volume of the micropores for H-MCM-41 with 10% Bindzil (E) compared to H-MCM-41 with 10% Bindzil (P). The difference was even lower between H-MCM-41 with 50% Bindzil (P) and H-MCM-41 with 50% Bindzil (E).

The median particle sizes determined by SEM and TEM were 66-111 nm and 32–44 nm, respectively (Fig. S1, S2 ESI† and Table 1) with the differences apparently related to the equipment resolution. For the Bindzil binder, the median particle sizes were smaller, but the width of the distribution was as broad as for H-MCM-41 and its agglomerates with a binder (Fig. S1, S2 ESI† and Table 1).

As expected, acidity was decreased with increasing concentration of the non-acidic binder (Table 1). Moreover, the measured values of Brønsted and Lewis acid sites were 26–68% lower than the theoretical ones. This was attributed to re-calcination of the catalyst after synthesis with a binder and after catalyst shaping, leading to formation of extra-framework alumina.^29,34^ A similar behaviour was also observed for Y and USY extrudates containing 70 wt% of pseudoboehmite or γ-Al_2_O_3_ as a binder.^33^

### 3.2 PFG NMR diffusion analysis

For all H-MCM-41 catalysts, experimental signal attenuations due to diffusion, *E*(*b*,Δ), measured using PFG NMR demonstrated a small deviation from a single-component Gaussian diffusion shape, *i.e.*, *E*(*b*,Δ) ≠ exp(– *D*(Δ)*b*). For example, Fig. 4a shows results for H-MCM-41 (M100-E) at different diffusion times Δ. Viewed in the logarithmic scale, there is an initial linear fast decay until about 10% of the initial signal strength, followed by an intermediate region and much slower linear-like decay when the signal decreases already by two orders of magnitude as *b* factor increases. This effect can be attributed either to presence of several porous sites, or to a complex manifestation of non-Gaussian diffusion in the restricted space of the catalysts. Depending on the porous structure, these effects can differ significantly,^35^ therefore various approaches are employed to analyze data.^5,9,35–37^ As the first approximation, average apparent diffusion coefficients (*D*(Δ)) were extracted by analyzing the slopes of very initial parts of these curves. Corresponding values obtained for H-MCM-41 (M100-E) are shown in Fig. 4b. Similar data for other samples are shown in ESI,† (Fig. S4–S12). In addition to this model-free approach, two other processing strategies (namely, two-component fitting and the Laplace inversion) that complement each other are also presented.

It was found that all studied H-MCM-41 containing materials exhibited only a mild dependence of the measured apparent diffusion coefficients *D*(Δ) on the diffusion time Δ, with only a slight decrease in the measured values as the diffusion time increases. For instance, Fig. 4b displays a characteristic example based on the data for H-MCM-41 (M100-E) catalyst. According to the analysis proposed by Mitra *et al.*,^25^ the surface-to-volume ratio (*S*/*V*) extracted using the initial slope of *D*(Δ) curve (Fig. S3b, ESI†) indicates geometrical constraints of 78 μm in size assuming spherical geometry which correlates with the size of used catalyst grains *d*_gr_ (below 63 μm) by the order of magnitude within the given experimental accuracy. Therefore, a slight decrease in the apparent diffusion coefficient could be attributed to the grain border effect, if reflective boundary conditions are met for the grain surface. The mechanism of the reflection, however, is unclear as both intragrain and intergrain spaces are filled with *n*-hexadecane in extrudates, hence, there can be other reasons for a slight alteration of the apparent diffusion coefficients with Δ. At the same time, it is clear that the mesoporous constraints are not the reason for such alteration at the applied diffusion times as the long-time limit for that pore sizes is met (4 nm ≪ 78 μm). Subsequently a rather averaged effect of the mesoporous/microporous medium inside the grains should be observed. The change in the diffusivities with Δ is very moderate (only *ca.* 3%, Fig. 4b), and in any case going from 20 to 240 ms diffusion time the attenuation curves reach stationary shapes. Therefore, it can be assumed that *D*(Δ) at the largest measured Δ = 240 ms is very close to the infinite time coefficient *D*(Δ → ∝) characterizing large diffusional displacements in the interconnected porous medium independent from the diffusion time. This stationary quantity gives the diffusivity averaged over diffusion inside microparticles and between them in a catalyst bed, and it is used as the effective diffusion coefficient *D*_e_ in the chemical engineering literature, Eqn (1).^17^ Therefore, it is assumed here that the attenuation curves *E*(*b*,Δ) at Δ = 240 ms generally characterize *D*_e_, which will be used in the following analysis.

**Fig. 5:**
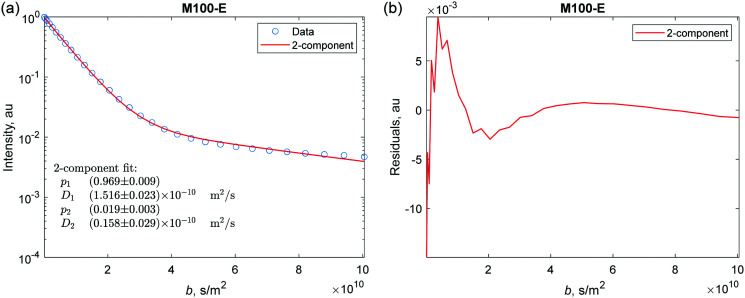
(a) The results of the two-component fit (red trace) of the spin-echo attenuation curves for Δ = 240 ms of H-MCM-41 (M100-E) catalyst. (b) Residuals resulting after the fitting procedure. Similar data for other samples are presented in the ESI.†

The experimentally measured *E*(*b*,Δ) curves can be viewed as a sum of two individual curves corresponding to a fast dominant diffusion component with a much smaller slow component. Therefore, the first type of analysis included a two-component fitting (Eqn (4)). H-MCM-41 materials generally may have anisotropic structure and one can use the known powder average models for anisotropic diffusion to analyze the current results.^9^ It should be noted, however, that due to small particle sizes of H-MCM-41 (see d_TEM_ and d_SEM_ in Table 1), measured diffusivities at the long-time limit correspond to an averaged transport over many particles, meaning that *n*-hexadecane spends significant time both in the isotropic medium between the particles and inside the anisotropic pores (roughly straight, long cylinders in an intact MCM-41 material) of the particles. One cannot expect any strong anisotropic diffusion effects, as the average between the two media is observed, thus the anisotropic diffusion analysis is not presented here. In addition to the two-component fitting, the Laplace inversion procedure was used as an approach to estimate distributions of the components based on the Gaussian diffusion assumption. The results are presented in the following sections.

As a characteristic example, Fig. 5a shows results for *E*(*b*,Δ = 240 ms) of two-component fitting (red curve) for H-MCM-41 (M100-E) extrudate. The main signal contribution for this sample represents the fast diffusion through the interconnected mesopores and the space between the particles in the grains, while there is only minor contribution of the slow diffusion component with the population fraction of only 0.019 (1.9%). Similar observations were acquired for other catalysts (see Fig. S4–S12 ESI†). Note that due to the low population of the slow diffusion component (commonly *p*_2_ ≈ 1–2%) it was difficult to perform reasonable fitting without introduction of weights into the fitting procedure. Weighted by the reciprocal signal intensity (1/*I*), the fitting produced a reasonable quality estimation for the slow component. The plot of residuals after the two-component fit is shown in (Fig. 5b).

**Fig. 6:**
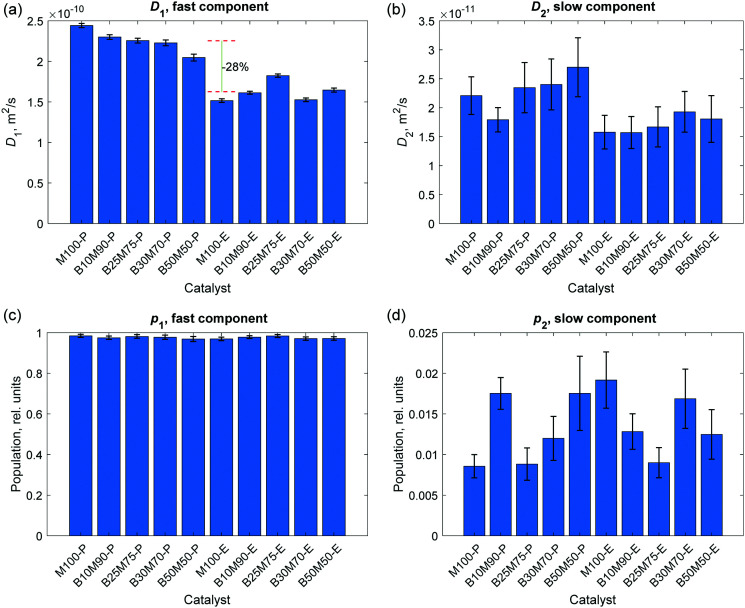
The results of the two-component fitting analysis of PFG NMR spin-echo attenuation curves measured for various mesoporous catalysts at diffusion time Δ = 240 ms. The bar charts represent apparent diffusion coefficients for the fast, *D*_1_, (a) and slow, *D*_2_, (b) diffusion components as well as for the populations of these components *p*_1_ (c) and *p*_2_ (d), respectively. The two dashed horizontal lines indicate diffusion coefficients averaged among powdered (upper red line) and extruded (lower red line) catalysts in (a). The difference between the averaged values (28%) is shown in percentage.

The results of the two-component fits for all catalysts at diffusion time Δ = 240 ms are summarized in Fig. 6. In general, a consistent decrease of the apparent diffusion coefficient for the fast (*D*_1_) diffusion component was observed when switching from the powder materials to the extrudates. The drop in the apparent diffusion coefficients is on the order of 30%. In the case of powdered samples M100-P – B50M50-P, there is also a slight tendency of slowing down the fast diffusion component (*D*_1_) as the concentration of the binder (Bindzil) increases from 0 to 50% (Fig. 6a), which might be reasonable, since the binder can create additional constrains in the medium. In contrast, the data for extrudates M100-E–B50M50-E do not reveal this trend, meaning that the diffusional transport in the extrudates is not very sensitive to the binder. At the same time, there are no dramatic changes in diffusivities between the powdered materials and extrudates.

The slow diffusion component does not show a very clear correlation with the shape of the material (powder or extrudate) within the obtained statistical confidence (Fig. 6b). This result indicates that a slow diffusion component may represent diffusion predominantly inside the porous microparticles. On the other hand, this can be simply an averaged effect of restricted diffusion generating non-Gaussian displacements in the confined porous matrix. At the same time, the two-component fitting analysis shows that the populations of slow and fast components do not change significantly from one catalyst to another, reflecting a strong domination of the fast component.

**Fig. 7:**
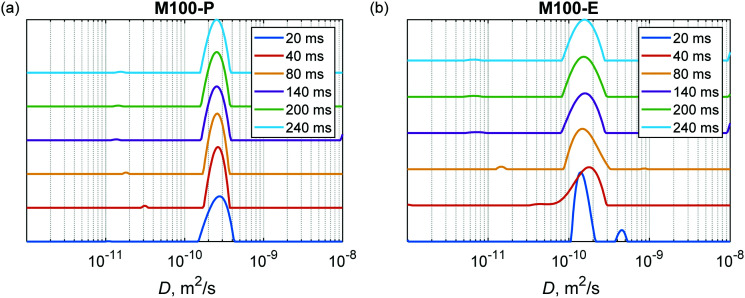
Diffusion coefficient distributions obtained in Laplace inversion of PFG NMR spin-echo attenuation curves at different diffusion times Δ (see legend) for (a) H-MCM-41 (M100-P) and (b) H-MCM-41 (M100-E) catalysts.

The Laplace inversion can provide estimated distributions of diffusion coefficients from the spin-echo attenuation curves measured in PFG NMR experiments. As an example, Fig. 7 shows estimated distributions for the powdered and extruded catalysts H-MCM-41 (M100-P) and H-MCM-41 (M100-E), respectively. Data for other samples are presented in the ESI† (Fig. S14–S17). In all cases, this analysis reveals a clear trend that the extrusion makes the diffusional transport slightly slower, which certainly correlates with the conclusions drown in the previous discussions.

These distributions also show that the major component in the case of powdered catalysts (*e.g.*, Fig. 7a) is not strongly dependent on the diffusion time Δ, meaning that this pool of *n*-hexadecane molecules is not sensitive to the confinement of the porous matrix. Most likely already at the shortest time Δ = 20 ms *n*-hexadecane molecules from the mesoporous and microporous sites are indistinguishable, showing only exchange averaged diffusional transport in porous particles. The root mean square displacement ((2*D*Δ)^1/2^) during the diffusion delay is on the order of several μm, which is much larger than the pore sizes. This dominant behaviour can be attributed to open unblocked pores. To the contrary, the minor component shows a decrease in the diffusion coefficient, which can be interpreted as pores with longer or/and narrower channels. As a comparison, Laplace inversion was also performed for Bindzil with *n*-pentadecane as a probe molecule in ref. 7 and in that case two distinguishable sites were observed with *D* values of 1 × 10^−10^ and 4 × 10^−10^ m^2^ s^−1^. In the case of *n*-hexadecane, the two components in Bindzil were not clearly resolved due to the lower diffusion coefficient, as the Laplace inversion procedure gave a broad distribution between 1 × 10^−10^ and 4 × 10^−10^ m^2^ s^−1^ for Δ ≥ 20 ms (Fig. S18, ESI†). The raw data for Bindzil are shown in Fig. S13 (ESI†).

Interestingly, in the case of extrudates (*e.g.*, Fig. 7b) at the shortest diffusion time Δ = 20 ms the second fast component is the one with the diffusion coefficient close to that of free diffusion. This component being systematically visible for all extrudates (Fig. 7b and Fig. S14b–S17b, ESI†), is not present for the powder samples. Therefore, one can assume that the fast component is not just an artefact arising from the experimental noise in the inversion procedure. It is likely that extrusion created a fraction of macropores where hexadecane can diffuse almost like in a free medium. It can be the large pores that were observed by SEM analysis (see the next section). With the diffusion time these two sites distinguishable at Δ = 20 ms merge, most likely as a result of the molecular exchange, and the averaged broader peak is observed for the joint pool of macro-, meso- and micropores. As in the case of powdered materials, some very minor component depending on the diffusion time was also observed which can correspond to pores with longer or/and narrower channels that are not exchanging *n*-hexadecane that fast.

**Fig. 8:**
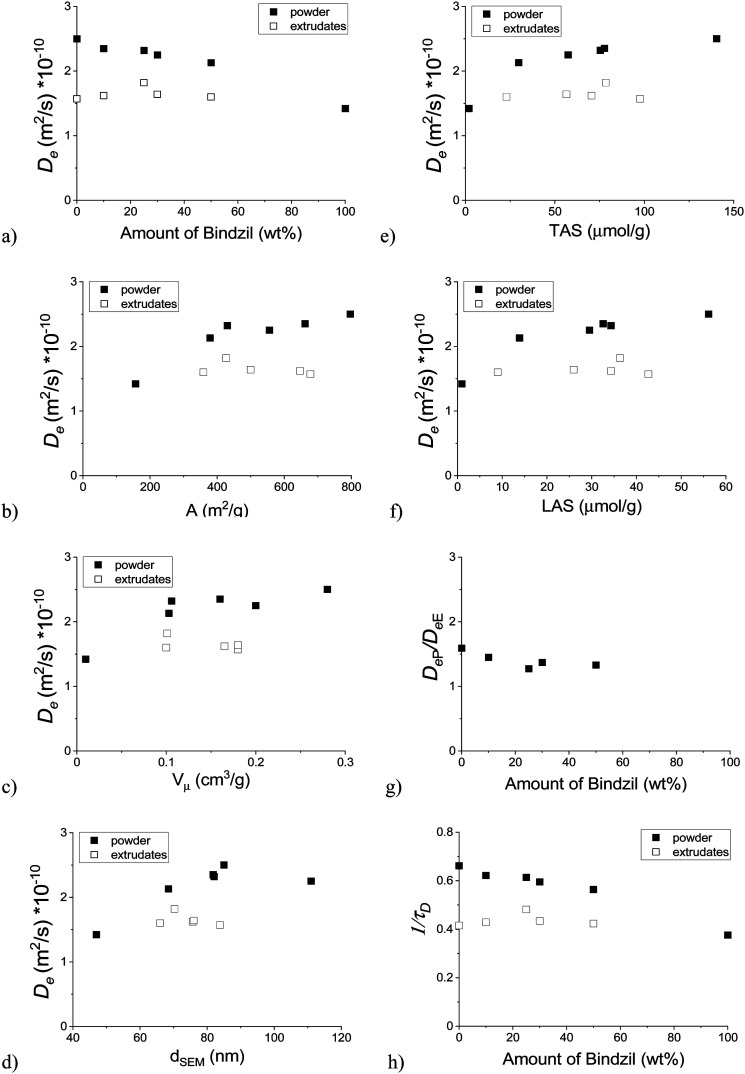
*D*_e_ at 240 ms as a function of: (a) the amount of Bindzil, (b) the specific surface area, (c) the micro-pore volume, (d) the particle size determined by SEM (e) the total acid sites, (f) the Lewis acid sites, (g) ratio of *D*_e_ coefficients for the powder and extrudates as a function of the amount of Bindzil, (h) 1/τ_D_ as a function of the amount of Bindzil.

Effective diffusion coefficients at the long diffusion time limit (Δ = 240 ms) are generally lower in extrudates than in powders (Fig. 8a) as was also observed for heptane and pentadecane diffusion in H-Beta-Bindzil catalysts.^7^ Diffusivities also decrease with an increasing amount of the Bindzil binder for powder catalysts, while for extrudates diffusivity was constant. An increase of the effective diffusion coefficient was observed with an increase of the specific surface area (Fig. 8b), and porosity, especially the micro-pore volume (Fig. 8c), the particle size (Fig. 8d) and also superficially with the amount of acid sites (Fig. 8e and f) in the case of powder catalysts. Diffusion of hexadecane controlled by the micropore diffusion is in line with the literature studies^6,7^ reporting heptane and pentadecane diffusion. Although it has been observed that diffusivity apparently increases with the Lewis, Brønsted, and overall acidity of the powder catalyst, it can only be a reflection of the textural properties, which, like acidity, varied with the amount of a binder (Table 1). This is in line with the previous observations^7,39^ suggesting that strong interactions of acidic zeolites with the hydrocarbons can lead to lower diffusivities,^38^ and, on the contrary, that heptane diffusivity increased with the increasing Lewis acidity.^7^ No correlation with Lewis or Brønsted acidity was observed for pentadecane diffusion.^7^

In the case of extrudates, no apparent correlation between the effective diffusion coefficient and the physico-chemical properties follows directly from Fig. 8. This could indicate that the transport pores in the extrudates have a stronger effect on the hexadecane diffusion than the micro-porosity or the catalyst acidity. Some visible large pores were observed by SEM analysis (Fig. 3), especially the large pores on the outmost layer of the extrudates were clearly seen (Fig. 3c). Another explanation can be attributed to the chemical interactions between the H-MCM-41 catalyst and the Bindzil binder leading to the heterogeneous distribution of the three phases (pristine H-MCM-41 catalyst, pristine Bindzil binder, and H-MCM-41-Bindzil agglomerates) in the shaped body of the catalyst. Such interactions were confirmed by SEM (Fig. 3) and by comparison of the observed and the theoretically predicted values of some physico-chemical properties (Table 1). These theoretical values were calculated as a linear combination of the contribution of non-agglomerated neat components in the powder form.

Fig. 8g shows that the ratio of the effective diffusion coefficient for the powder catalyst to the extrudates decreased with the increasing amount of the Bindzil binder in the sample. A small deviation from the visible trend for the catalysts containing 25–30 wt% of the Bindzil binder can be attributed to chemical interactions as explained above.

When calculating the coefficient 1/*τ*_*D*_ (Eqn 1) using the effective diffusion coefficient *D*_e_ and the self-diffusion coefficient for *n*-hexadecane in the bulk *D*_0_ = 3.78 × 10^−10^ m^2^ s^−1^ at 25 °C and 1 bar (ref. 39 and also measured in this work) and plotting this as a function of the amount of Bindzil (Fig. 8h), it can be observed that for the extrudates these values vary in the range of 0.42–0.48 being independent from the amount of Bindzil. On the contrary, for the powder catalysts 1/*τ*_*D*_ are dependent on the binder content, being much higher, in the range of 0.38–0.66, as expected. When comparing these data with those in ref.7 for pentadecane diffusion obtained in Bindzil(P) for which 1/*τ*_*D*_ = 0.40, it can be observed that for hexadecane this value, being 0.38, was very close. When pentadecane diffusion was studied in microporous H-Beta-Bindzil powder, 1/*τ*_*D*_ varied from 0.60–0.68. For extrudates with 30% Bindzil the 1/*τ*_*D*_ value of 0.56 was reported, similar to the current case of hexadecane and the powder catalyst. These values for extrudates for pentadecane and hexadecane were 0.56, ref.,^7^ and 0.42 being closer to each other.

From Fig. 8h it clearly follows that the effective diffusion coefficient and 1/τ_D_ is lower for the pristine binder compared to the mesoporous material *per se*. Some textural properties (Table 1) for the powder catalysts were apparently not linearly scaling with the binder content, with the deviations from linearity also visible in Fig. 8a and h. This implies already some interactions between the binder and the mesoporous MCM-41 when the powder for subsequent extrusion was prepared. The preparation procedure included using several steps with a mechanical impact being the reason for changes in the textural properties. Interestingly enough, after extrusion the diffusivity decreased suggesting blocking of some micropores and thus lower effective diffusivity through the catalyst body. The influence of the binder is, however, more complex. The negative effects of a binder (*e.g.*, alumina) on porosity and thus on the mass transfer was connected in the literature^40^ with the blocking the micropores in zeolites. This should lead to a decrease in effective diffusivity with an increase in the binder content for the extrudates from 0 to 30%. An alternative observation^2^ implies more transport pores upon addition of the binder and higher effective diffusivity when the binder content is increasing. Some indications of this behaviour could be seen from Fig. 8a, even if, as mentioned above, the changes are not profound. In any case, the magnitude of the increase was not sufficient to reach the corresponding value for a mixture of MCM-41 and the binder in a powder form.

## 4. Conclusions

A comparison of diffusion coefficients for *n*-hexadecane was performed in mesoporous H-MCM-41 powder and extrudate catalysts containing different amounts of silica (Bindzil) as a binder. Several H-MCM-41 catalysts with different amounts of Bindzil as a binder were shaped by extrusion. In addition, mechanical mixtures of H-MCM-41 and the binder at different ratios were studied. A PFG NMR method was applied to determine diffusivity of *n*-hexadecane in all catalysts. The parent powder form H-MCM-41 exhibits 1 nm pores and specific surface area of *ca.* 800 m^2^ s^−1^, while the extrudates possess slightly smaller specific surface areas. When introducing different amounts of Bindzil, the specific surface area decreased with an increasing amount of Bindzil, except for 30% Bindzil and it was also smaller for extrudates in comparison to the powder catalysts. Acidity of the catalysts decreased as expected with an increasing amount of the non-acidic binder.

Diffusion coefficients were measured in all catalysts applying the PFG NMR technique. The effective diffusion coefficient *D*_e_ increased for the powder catalysts with an increasing amount of the binder, while it remained constant for extrudates. Furthermore, the apparent diffusion coefficient was 30% higher for the powder catalysts than for the extrudates indicating hindered diffusion in extrudates. The coefficient connecting *D*_e_ and *D*_0_ was *ca.* 0.4 for hexadecane diffusion in extrudates, while it was *ca.* 0.6 for the powder catalysts indicating better mass transport in the latter. The effective diffusion coefficients *D*_e_ are important for modelling catalytic processes in reactors, therefore these results provide valuable insights clearly showing how the complex extrudate structure can affect the catalytic behaviour.

## Conflicts of interest

There are no conflicts of interest to declare.

## Supplementary Material

CP-024-D2CP00138A-s001
