# Supplementary material for: Diffusion measurements of hydrocarbons in H-MCM-41 extrudates with pulsed-field gradient nuclear magnetic resonance spectroscopy
Source: Phys Chem Chem Phys. 2022 Mar 16;24(14):8269–78. doi: 10.1039/d2cp00138a (PMC8985658; doi:10.1039/d2cp00138a)
Supplement: CP-024-D2CP00138A-s001 [file CP-024-D2CP00138A-s001.pdf]

Electronic Supplementary Material (ESI) for Physical Chemistry Chemical Physics.
This journal is © the Owner Societies 2022

Supporting Information

Diffusion measurements of hydrocarbons in H-MCM-41 extrudates with pulsed-field gradient nuclear magnetic resonance spectroscopy

Vladimir V. Zhivonitko[a], Zuzana Vajglová[b], Päivi Mäki-Arvela[b], Narendra Kumar[b], Markus Peurla[c], Ville-Veikko Telkki[1] and Dmitry Yu. Murzin[b*]

[a] *NMR Research Unit, University of Oulu, Oulu, Finland*

[b] *Åbo Akademi University, Johan Gadolin Process Chemistry Centre, Henriksgatan 2, Turku/Åbo, Finland, 20500*

[c] *Institute of Biomedicine, University of Turku, Kiinamyllynkatu 10, Turku, Finland, 20520*

[*] *dmurzin@abo.fi, corresponding author*

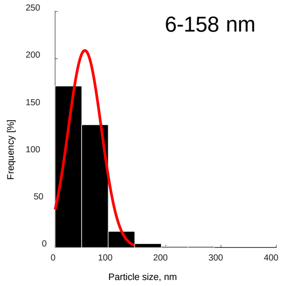
6-158 nm
Frequency [%]
Particle size, nm

a)

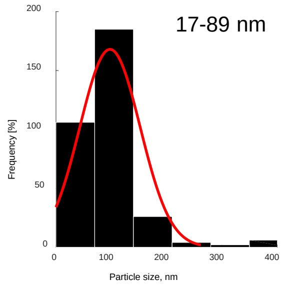
17-89 nm
Frequency [%]
Particle size, nm

b)

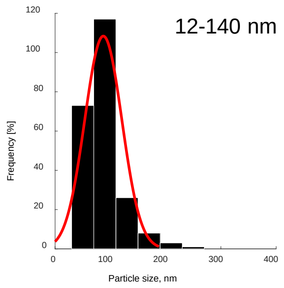
12-140 nm
Frequency [%]
Particle size, nm

c)

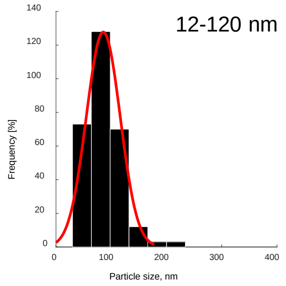
12-120 nm
Frequency [%]
Particle size, nm

d)

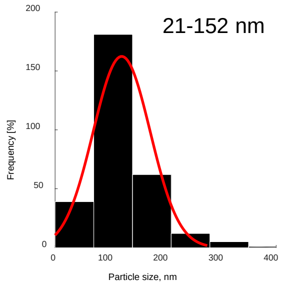
21-152 nm
Frequency [%]
Particle size, nm

e)

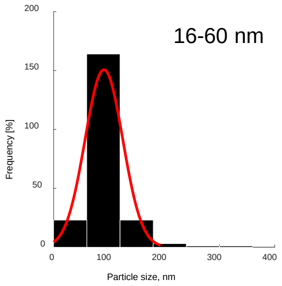
16-60 nm
Frequency [%]
Particle size, nm

g)

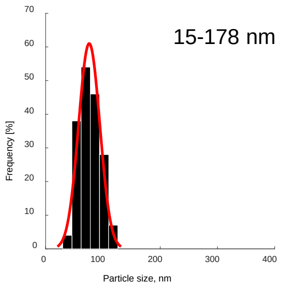
15-178 nm
Frequency [%]
Particle size, nm

h)

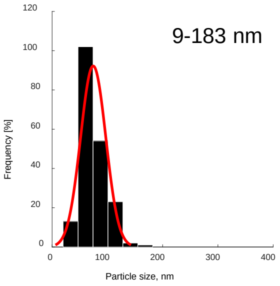
9-183 nm
Frequency [%]
Particle size, nm

i)

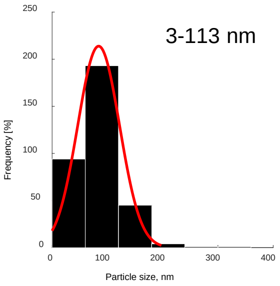
3-113 nm
Frequency [%]
Particle size, nm

j)

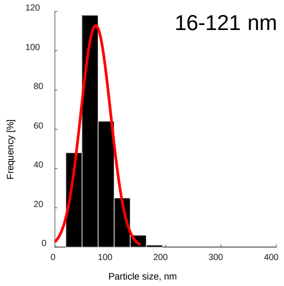

f)

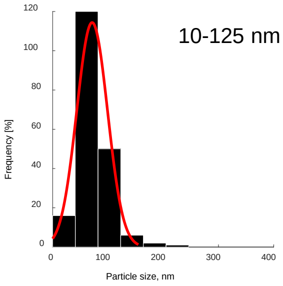

k)

Fig. S1 Particle size distribution determined from SEM: a) Bindzil (**B100-P**), b) H-MCM-41 (**M100-P**), c) H-MCM-41 with 10% Bindzil (**B10M90-P**), d) H-MCM-41 with 25% Bindzil (**B25M75-P**), e) H-MCM-41 with 30% Bindzil (**B30M70-P**), f) H-MCM-41 with 50% Bindzil (**B50M50-P**), g) H-MCM-41 (**M100-E**), h) H-MCM-41 with 10% Bindzil (**B10M90-E**), i) H-MCM-41 with 25% Bindzil (**B25M75-E**), j) H-MCM-41 with 30% Bindzil (**B30M70-E**), k) H-MCM-41 with 50% Bindzil (**B50M50-E**).

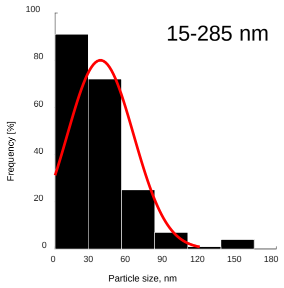
15-285 nm
Frequency [%]
Particle size, nm

a)

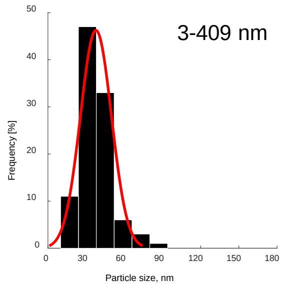
3-409 nm
Frequency [%]
Particle size, nm

b)

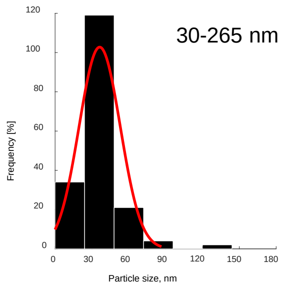
30-265 nm
Frequency [%]
Particle size, nm

c)

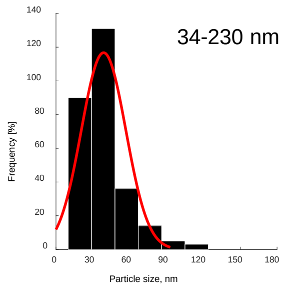
34-230 nm
Frequency [%]
Particle size, nm

d)

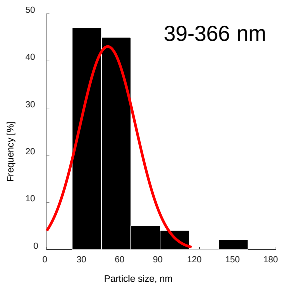
39-366 nm
Frequency [%]
Particle size, nm

e)

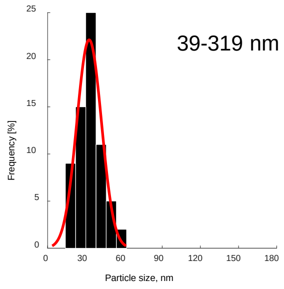
39-319 nm
Frequency [%]
Particle size, nm

g)

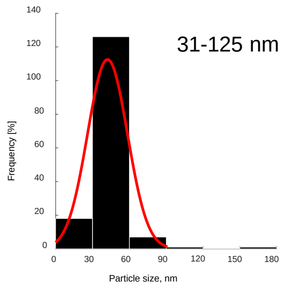
31-125 nm
Frequency [%]
Particle size, nm

h)

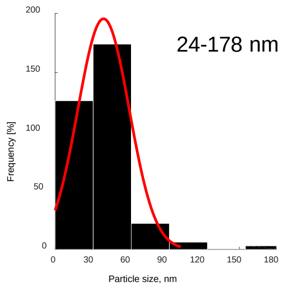
24-178 nm
Frequency [%]
Particle size, nm

i)

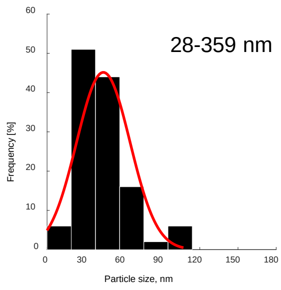
28-359 nm
Frequency [%]
Particle size, nm

j)

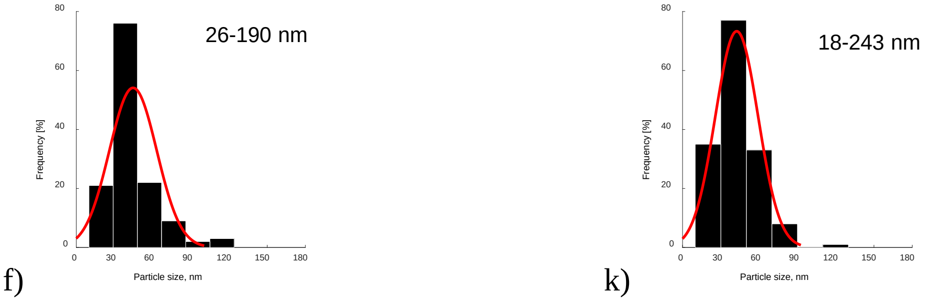

Fig. S2 Particle size distribution determined from TEM: a) Bindzil (**B100-P**), b) H-MCM-41 (**M100-P**), c) H-MCM-41 with 10% Bindzil (**B10M90-P**), d) H-MCM-41 with 25% Bindzil (**B25M75-P**), e) H-MCM-41 with 30% Bindzil (**B30M70-P**), f) H-MCM-41 with 50% Bindzil (**B50M50-P**), g) H-MCM-41 (**M100-E**), h) H-MCM-41 with 10% Bindzil (**B10M90-E**), i) H-MCM-41 with 25% Bindzil (**B25M75-E**), j) H-MCM-41 with 30% Bindzil (**B30M70-E**), k) H-MCM-41 with 50% Bindzil (**B50M50-E**).

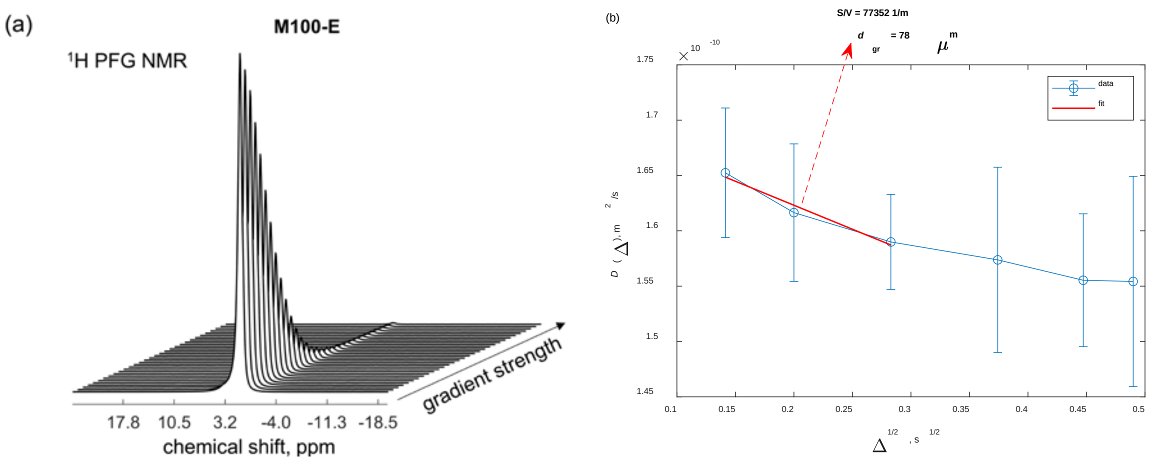

Fig. S3 (a) ^{1}H PFG NMR spectra as a function of the gradient strength measured for n-hexadecane in H-MCM-41 (**M100-E**) at diffusion time Δ = 240 ms. (b) Estimation of S/V ratio from the initial slope of D(Δ) values and the corresponding grain size d_{gr}.

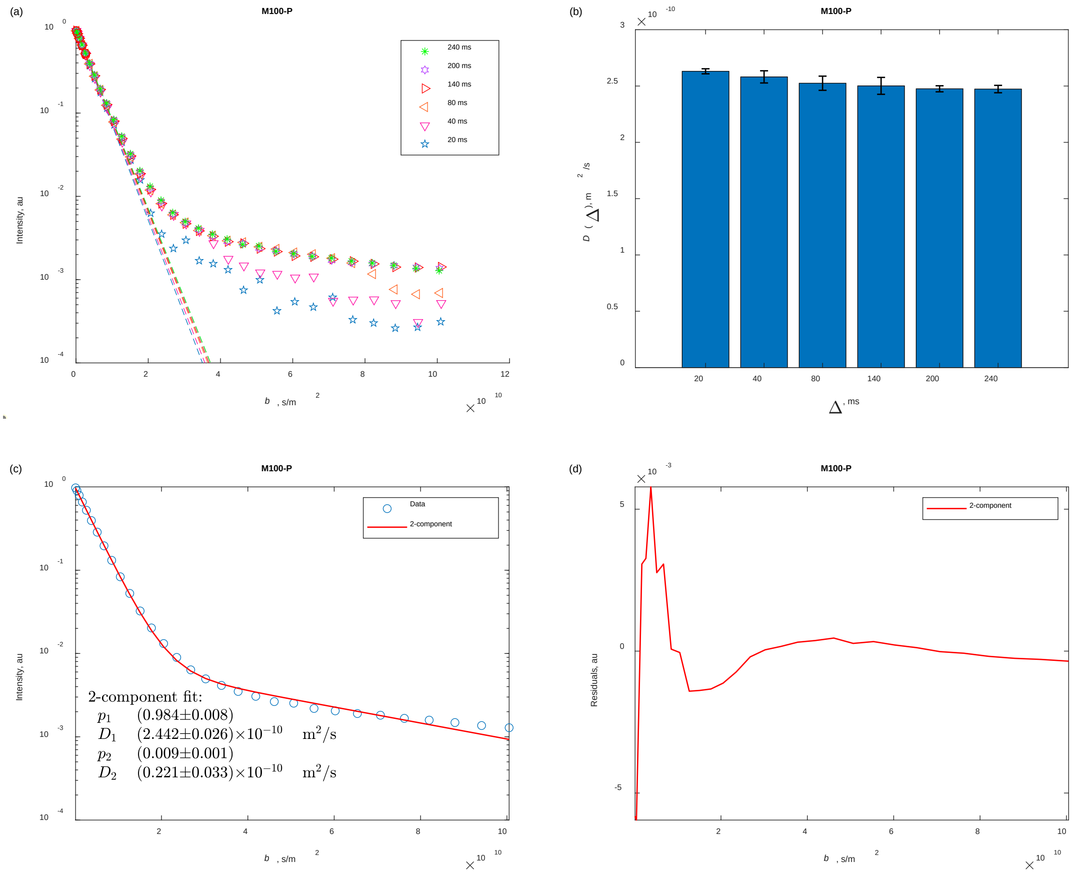

Fig. S4 (a) Spin-echo attenuation curves at different diffusion times Δ (see legend) obtained for n-hexadecane in H-MCM-41 (**M100-P**). The straight lines show the initial slope of the curves. (b) Apparent diffusion coefficient values extracted from the initial slope data. (c) Results of the 2-component fit (red curve) of the spin-echo attenuation curves for Δ = 240 ms of the same catalyst. (d) Residuals resulting after the fitting procedure.

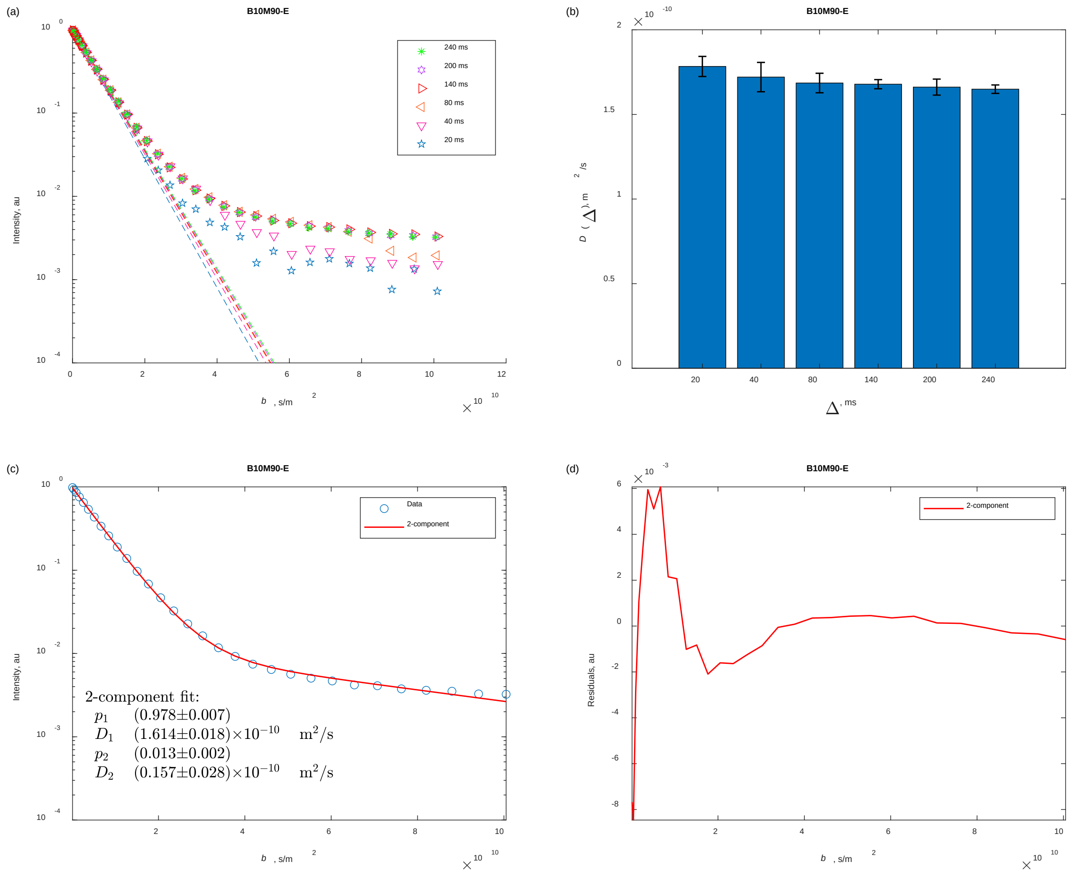

Fig. S5 (a) Spin-echo attenuation curves at different diffusion times Δ (see legend) obtained for n-hexadecane in H-MCM-41 with 10% of Bindzil (**B10M90-E**). The straight lines show the initial slope of the curves. (b) Apparent diffusion coefficient values extracted from the initial slope data. (c) Results of the 2-component fit (red curve) of the spin-echo attenuation curves for Δ = 240 ms of the same catalyst. (d) Residuals resulting after the fitting procedure.

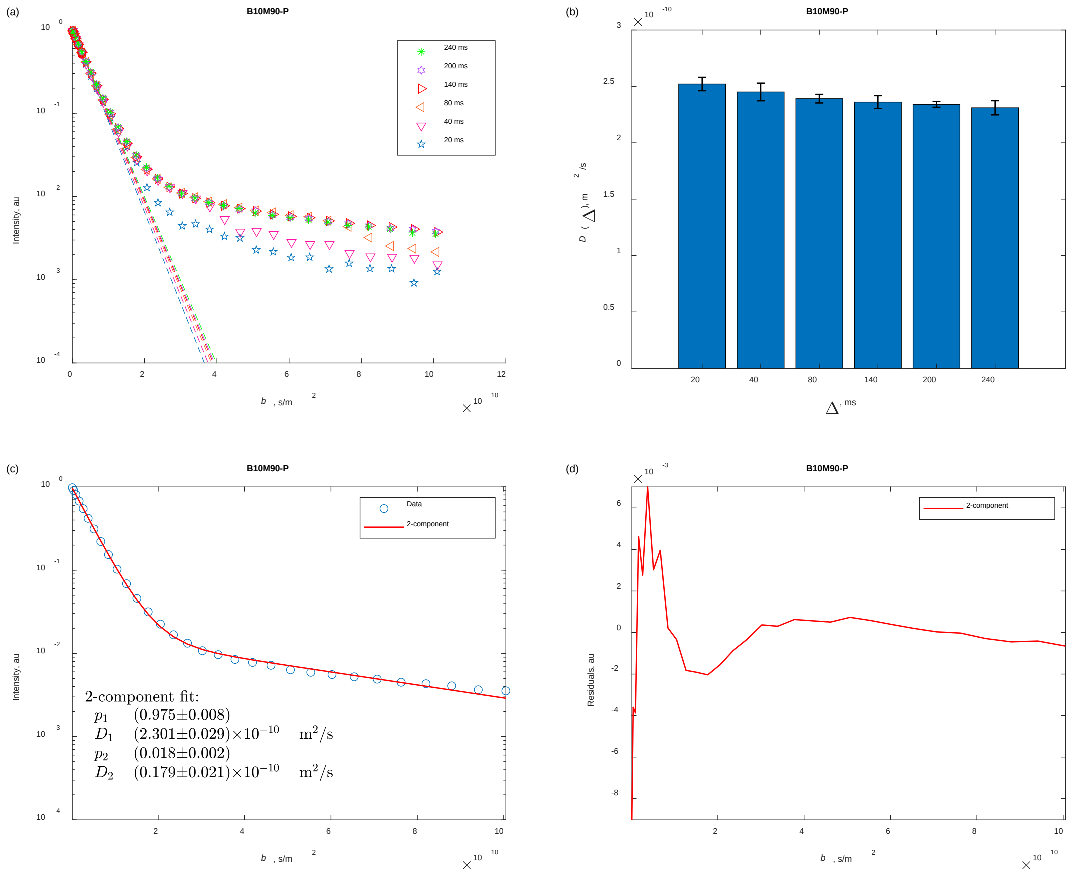

Fig S6 (a) Spin-echo attenuation curves at different diffusion times Δ (see legend) obtained for n-hexadecane in H-MCM-41 with 10% of Bindzil (**B10M90-P**). The straight lines show the initial slope of the curves. (b) Apparent diffusion coefficient values extracted from the initial slope data. (c) Results of the 2-component fit (red curve) of the spin-echo attenuation curves for Δ = 240 ms of the same catalyst. (d) Residuals resulting after the fitting procedure.

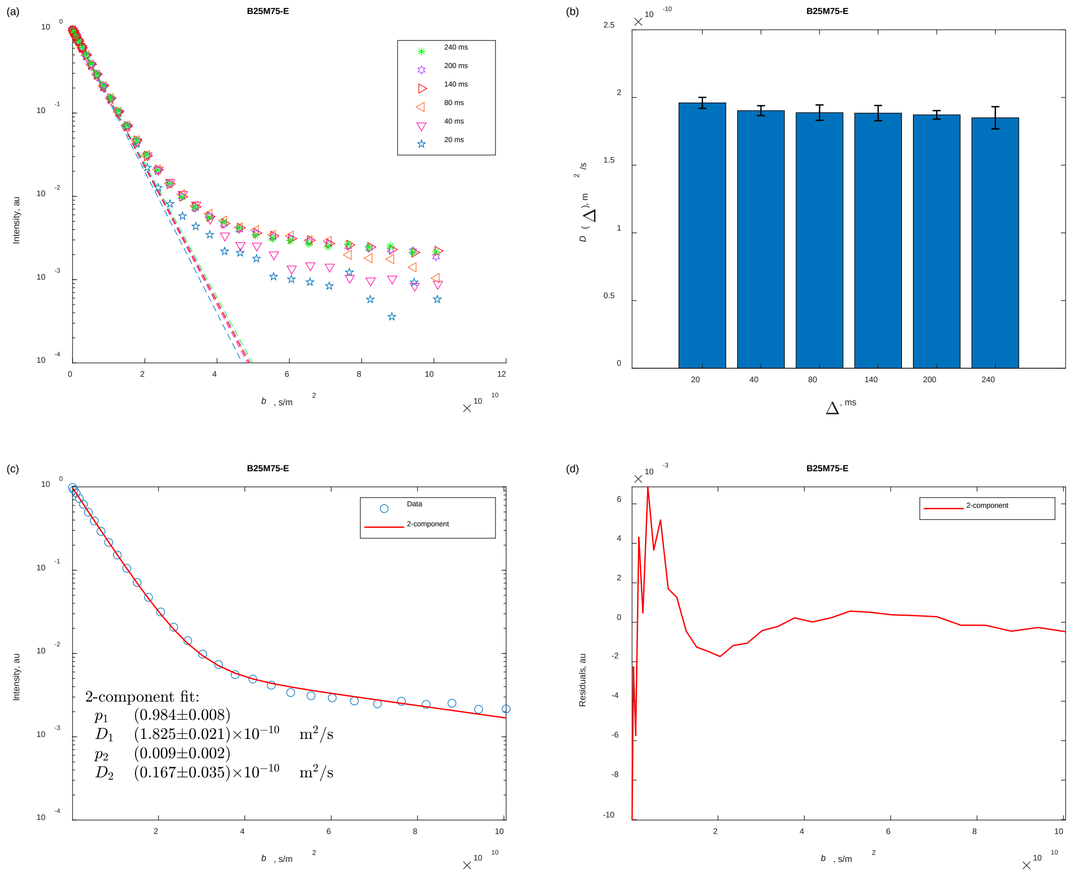

Fig. S7 (a) Spin-echo attenuation curves at different diffusion times Δ (see legend) obtained for n-hexadecane in H-MCM-41 with 25% of Bindzil (**B25M75-E**). The straight lines show the initial slope of the curves. (b) Apparent diffusion coefficient values extracted from the initial slope data. (c) Results of the 2-component fit (red curve) of the spin-echo attenuation curves for Δ = 240 ms of the same catalyst. (d) Residuals resulting after the fitting procedure.

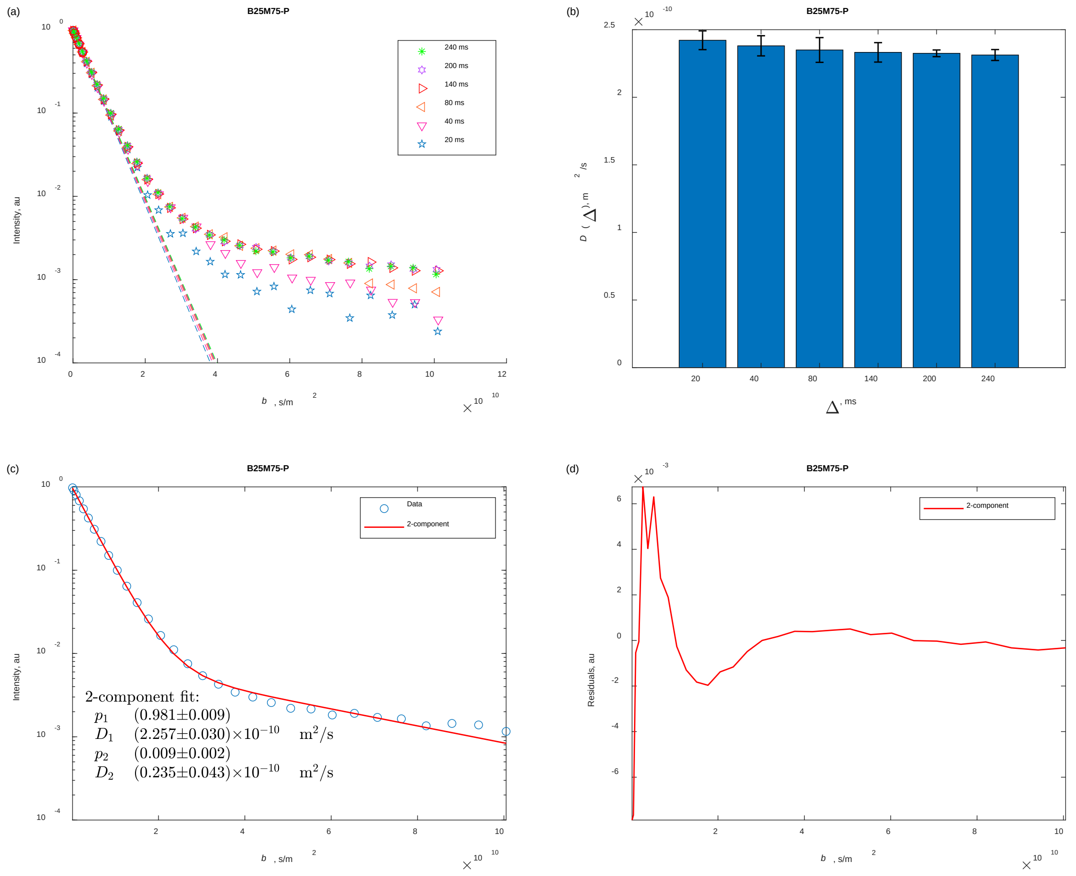

Fig. S8 (a) Spin-echo attenuation curves at different diffusion times Δ (see legend) obtained for n-hexadecane in H-MCM-41 with 25% of Bindzil (**B25M75-P**). The straight lines show the initial slope of the curves. (b) Apparent diffusion coefficient values extracted from the initial slope data. (c) Results of the 2-component fit (red curve) of the spin-echo attenuation curves for Δ = 240 ms of the same catalyst. (d) Residuals resulting after the fitting procedure.

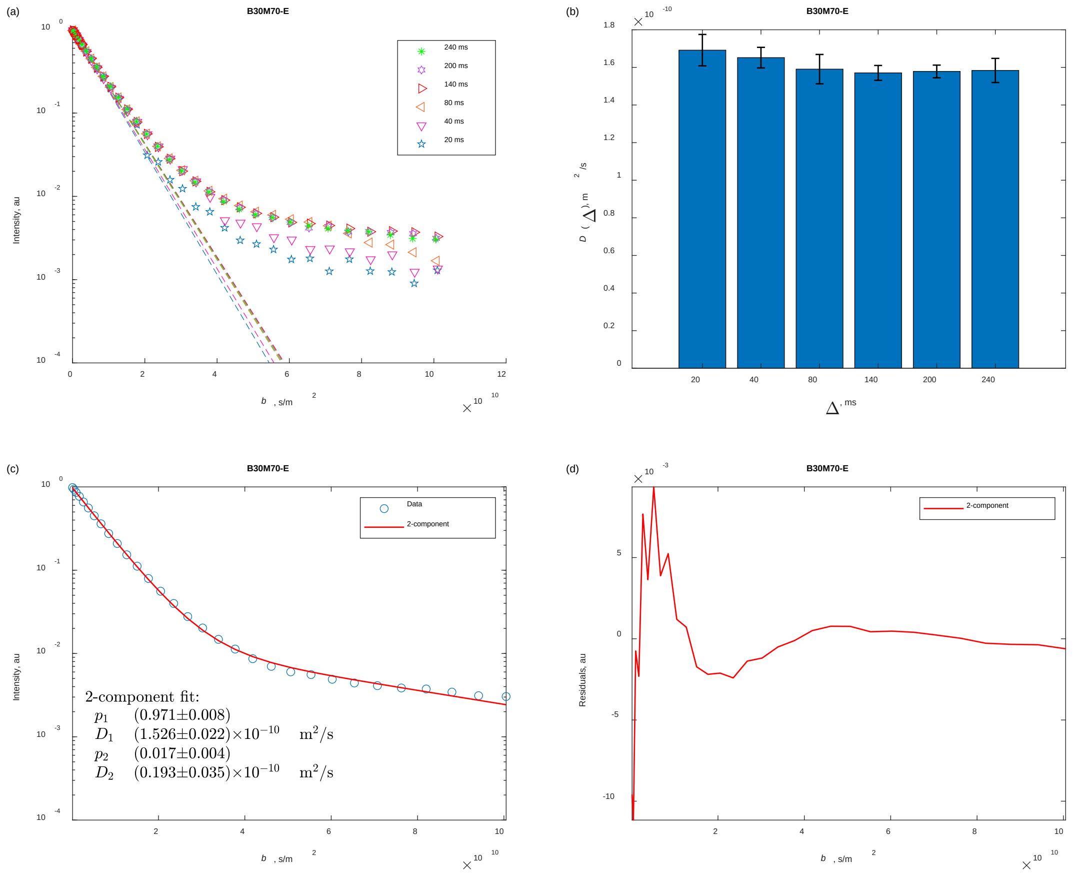

Fig. S9 (a) Spin-echo attenuation curves at different diffusion times Δ (see legend) obtained for n-hexadecane in H-MCM-41 with 30% of Bindzil (**B30M70-E**). The straight lines show the initial slope of the curves. (b) Apparent diffusion coefficient values extracted from the initial slope data. (c) Results of the 2-component fit (red curve) of the spin-echo attenuation curves for Δ = 240 ms of the same catalyst. (d) Residuals resulting after the fitting procedure.

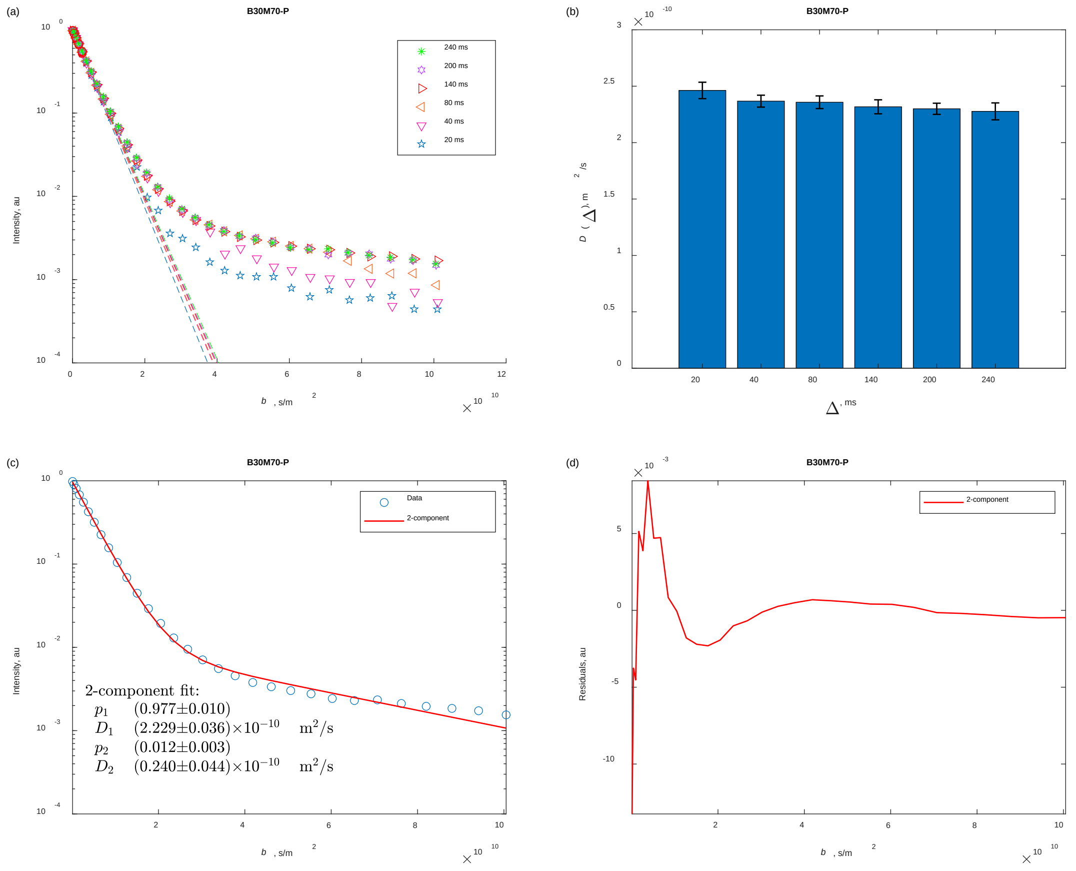

Fig. S10 (a) Spin-echo attenuation curves at different diffusion times Δ (see legend) obtained for n-hexadecane in H-MCM-41 with 30% of Bindzil (**B30M70-P**). The straight lines show the initial slope of the curves. (b) Apparent diffusion coefficient values extracted from the initial slope data. (c) Results of the 2-component fit (red curve) of the spin-echo attenuation curves for Δ = 240 ms of the same catalyst. (d) Residuals resulting after the fitting procedure.

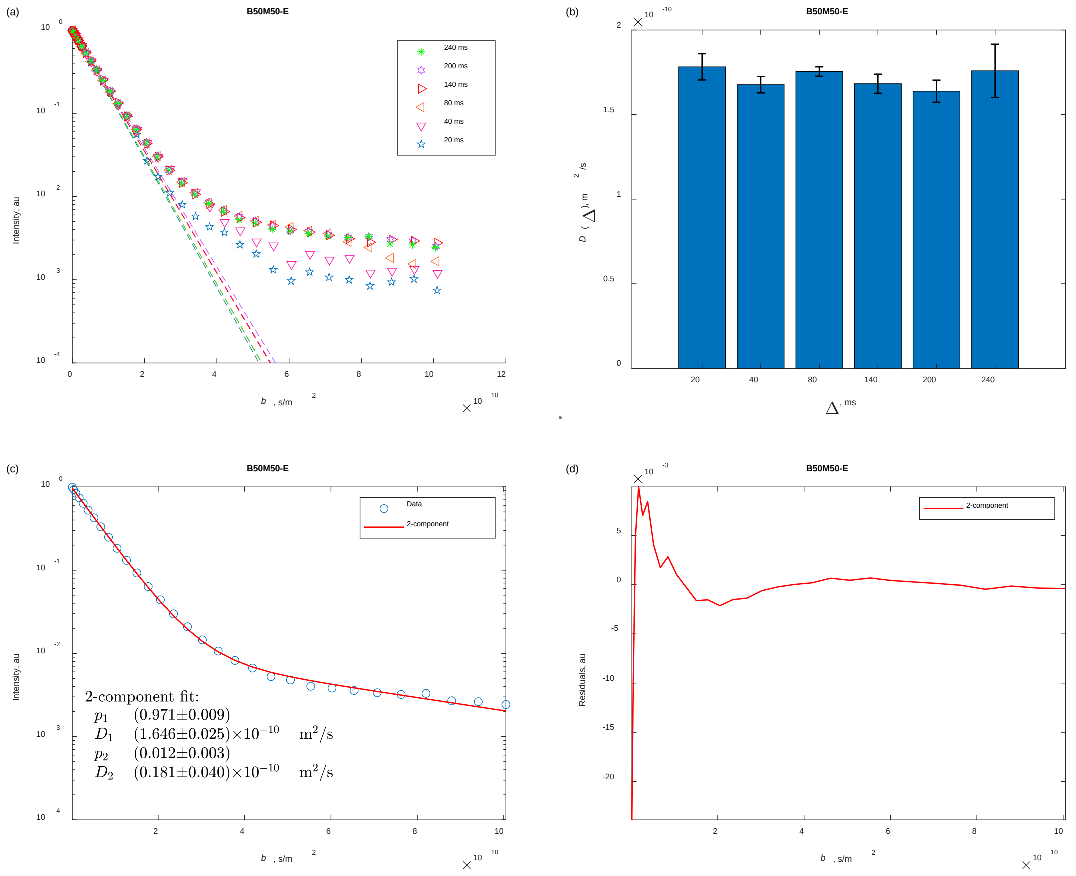

Fig. S11 (a) Spin-echo attenuation curves at different diffusion times Δ (see legend) obtained for n-hexadecane in H-MCM-41 with 50% of Bindzil (**B50M50-E**). The straight lines show the initial slope of the curves. (b) Apparent diffusion coefficient values extracted from the initial slope data. (c) Results of the 2-component fit (red curve) of the spin-echo attenuation curves for Δ = 240 ms of the same catalyst. (d) Residuals resulting after the fitting procedure.

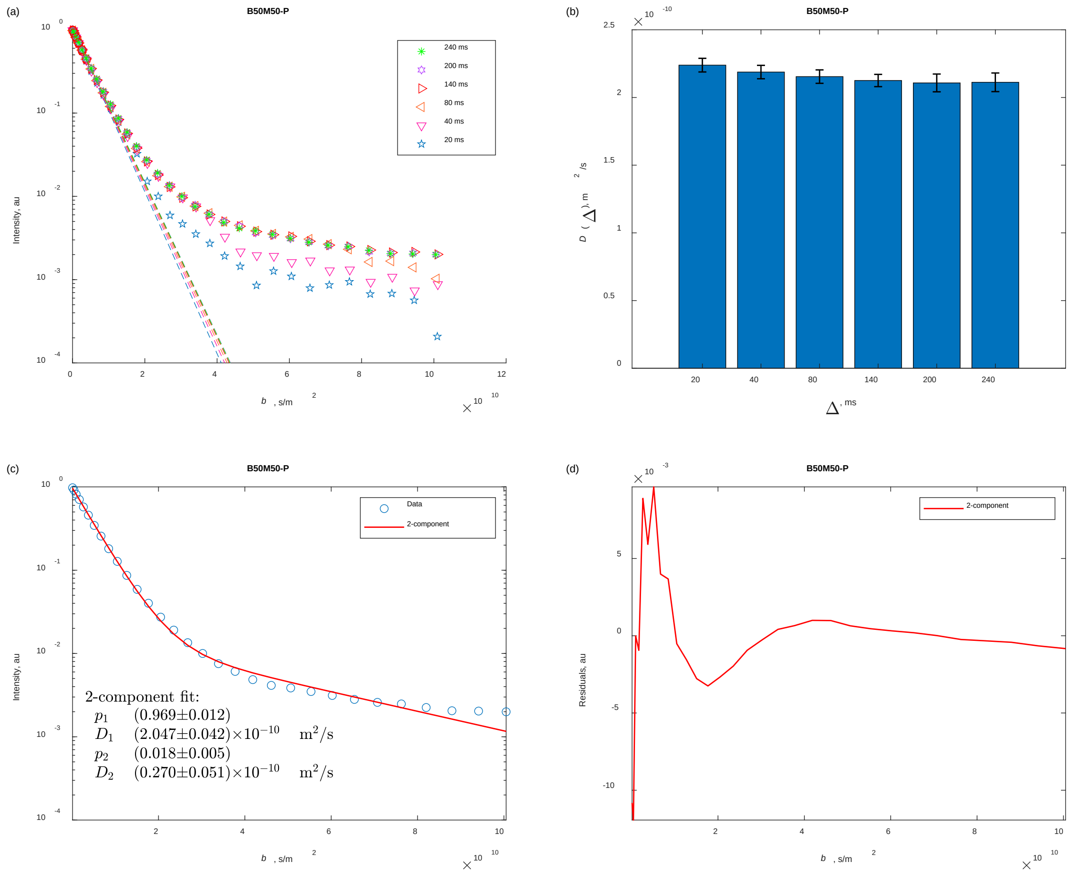

Fig. S12 (a) Spin-echo attenuation curves at different diffusion times Δ (see legend) obtained for n-hexadecane in H-MCM-41 with 50% of Bindzil (**B50M50-P**). The straight lines show the initial slope of the curves. (b) Apparent diffusion coefficient values extracted from the initial slope data. (c) Results of the 2-component fit (red curve) of the spin-echo attenuation curves for Δ = 240 ms of the same catalyst. (d) Residuals resulting after the fitting procedure.

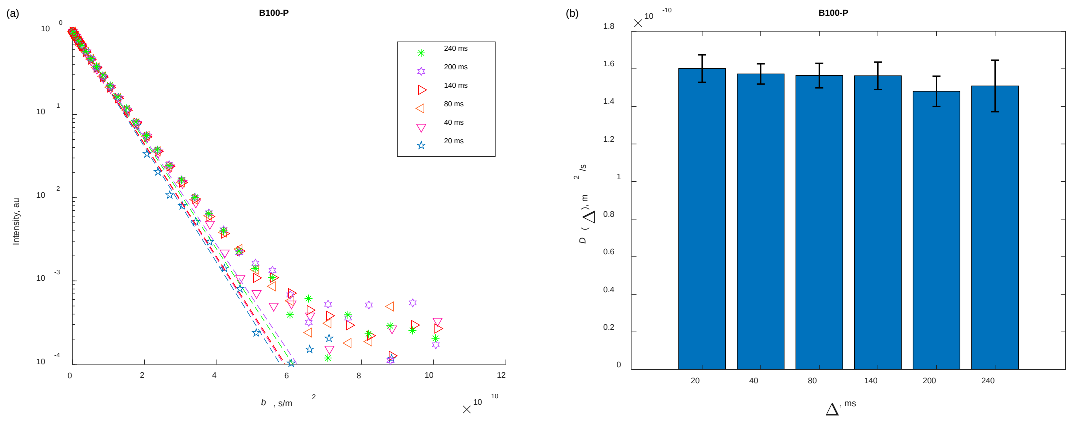

Fig. S13 Spin-echo attenuation curves at different diffusion times Δ (see legend) obtained for n-hexadecane in Bindzil (**B100-P**). The straight lines show the initial slope of the curved. (b) Apparent diffusion coefficient values extracted from the initial slope data.

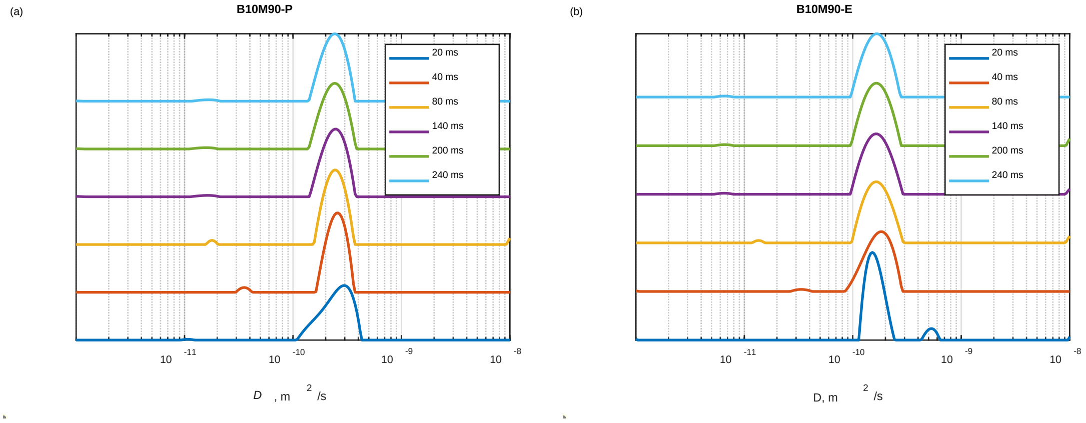

Fig. S14 Diffusion coefficient distributions obtained in Laplace inversion of PFG NMR spin-echo attenuation curves at different diffusion times Δ (see legend) for (a) H-MCM-41 with 10% of Bindzil (**B10M90-P**) and (b) H-MCM-41 with 10% of Bindzil (**B10M90-E**) catalysts.

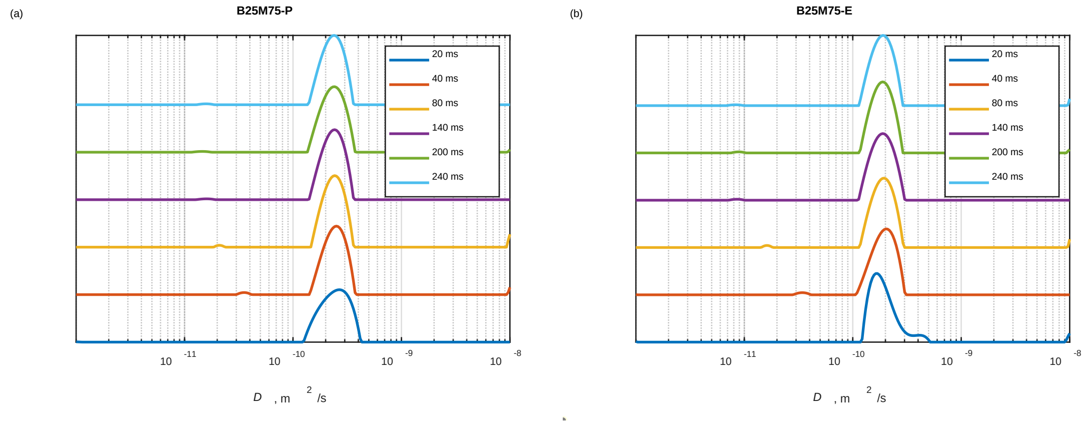

Fig. S15 Diffusion coefficient distributions obtained in Laplace inversion of PFG NMR spin-echo attenuation curves at different diffusion times Δ (see legend) for (a) H-MCM-41 with 25% of Bindzil (**B25M75-P**) and (b) H-MCM-41 with 25% of Bindzil (**B25M75-E**) catalysts.

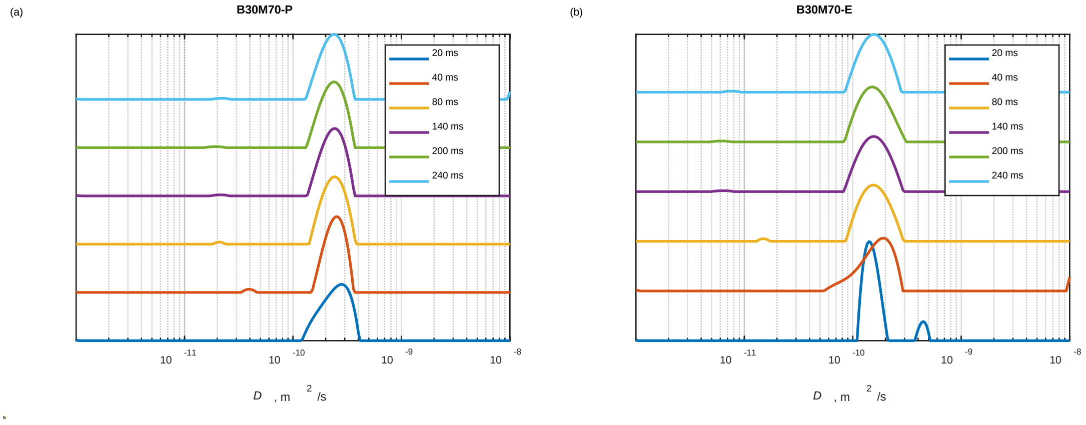

Fig. S16 Diffusion coefficient distributions obtained in Laplace inversion of PFG NMR spin-echo attenuation curves at different diffusion times Δ (see legend) for (a) H-MCM-41 with 30% of Bindzil (**B30M70-P**) and (b) H-MCM-41 with 30% of Bindzil (**B30M70-E**) catalysts.

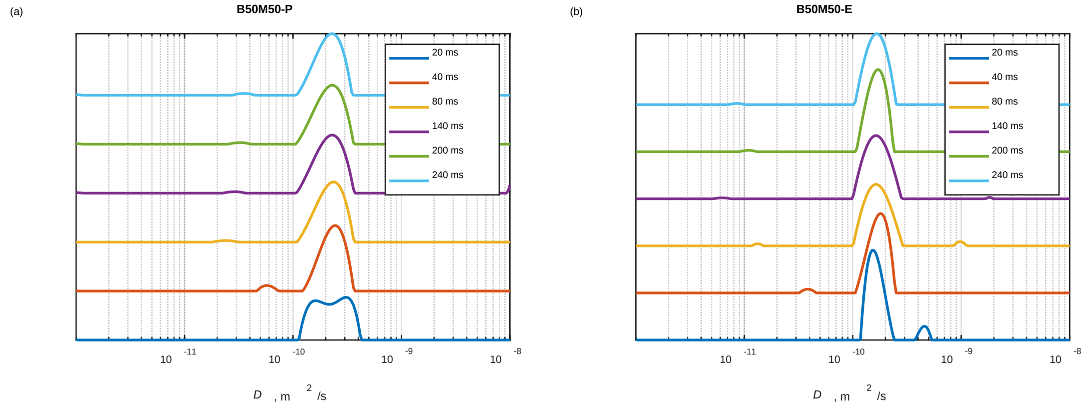

Fig. S17 Diffusion coefficient distributions obtained in Laplace inversion of PFG NMR spin-echo attenuation curves at different diffusion times Δ (see legend) for (a) H-MCM-41 with 50% of Bindzil (**B50M50-P**) and (b) H-MCM-41 with 50% of Bindzil (**B50M50-E**) catalysts.

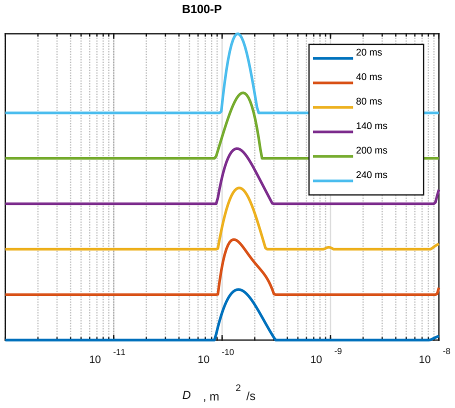

Fig. S18 Diffusion coefficient distributions obtained in Laplace inversion of PFG NMR spin-echo attenuation curves at different diffusion times Δ (see legend) for Bindzil (**B100-P**).
